# Supplementation of a Standardized Extract from *Phyllanthus emblica* Improves Cardiovascular Risk Factors and Platelet Aggregation in Overweight/Class-1 Obese Adults

**DOI:** 10.1089/jmf.2014.0178

**Published:** 2015-04-01

**Authors:** Savita Khanna, Amitava Das, James Spieldenner, Cameron Rink, Sashwati Roy

**Affiliations:** Department of Surgery, The Ohio State University Wexner Medical Center, Columbus, Ohio, USA.

**Keywords:** *cardiovascular disease*, *high-sensitivity C-reactive protein*, Phyllanthus emblica, *platelet aggregation*

## Abstract

The objective of this study (clinicaltrials.gov NCT01858376) was to determine the effect of oral supplementation of a standardized extract of *Phyllanthus emblica* (CAPROS^®^) on cardiovascular disease (CVD) risk factors in overweight adult human subjects from the US population. Overweight/Class-1 obese (body–mass index: 25–35) adult subjects received 500 mg of CAPROS supplement b.i.d for 12 weeks. The study design included two baseline visits followed by 12 weeks of supplementation and then 2 weeks of washout. At all visits, peripheral venous blood was collected in sodium citrate tubes. Lipid profile measurements demonstrated a significant decrease in calculated low-density lipoprotein cholesterol and total cholesterol/high-density lipoprotein following 12 weeks of CAPROS supplementation when compared to averaged baseline visits. Circulatory high-sensitivity C reactive protein (hs-CRP) levels were significantly decreased after 12 weeks of supplementation. In addition, both ADP- and collagen-induced platelet aggregation was significantly downregulated following 12 weeks of supplementation. Overall, the study suggests that oral CAPROS supplementation may provide beneficial effects in overweight/Class-1 obese adults by lowering multiple global CVD risk factors.

## Introduction

There is a growing interest and awareness of the role of dietary supplements and herbal medicine in the treatment and prevention of cardiovascular disease (CVD).^1^ Much of such interest centers on the use of herbal medicines with antioxidant properties that may lower conventional risk factors as well as may have antithrombotic effects.^1^ *Phyllanthus emblica* L. (*Euphorbiaceae*) (also known as amla or Indian gooseberry) is an edible fruit indigenous to tropical India and southeast Asia. Amla has been used for thousands of years in the traditional Indian system of medicine for the treatment of several diseases such as hemorrhage, diarrhea, jaundice, and dyspepsia.^2,3^ *Phyllanthus emblica*, traditionally believed to be an important source of vitamin C, has more recently been shown to contain low-molecular-weight hydrolyzable tannoids, including Emblicanin-A, Emblicanin-B, Punigluconin and Pedunculagin.^4–6^ Furthermore, this plant contains phenolic compounds, tannins, phyllembelic acid, phyllembelin, rutin, curcuminoids, and emblicol.^5^ Multiple studies have demonstrated the antioxidant properties of amla in a variety of systems.^7–10^ CAPROS^®^ is a standardized aqueous extract of the edible fruit of *Phyllanthus emblica* (amla) containing about 60% of low-molecular-weight hydrolyzable tannins such as Emblicanin-A, Emblicanin-B, Punigluconin, and Pedunculagin.^11^ Recently, studies on Indian population investigating the effect of CAPROS on endothelial cell dysfunction and platelet aggregation in subjects with type 2 diabetes reported a significant decrease in lipid and blood glucose levels, as well as inhibition of platelet aggregation.^11–13^ The current study sought to determine the effect of oral supplementation of CAPROS on CVD risk factors, including hyperlipidemia, C reactive protein (CRP), and high- sensitivity C reactive protein (hs-CRP) levels, and platelet aggregation in overweight/Class-1 obese adult human subjects from the US population.

## Materials and Methods

### Study subjects and experimental design

The study protocol (clinicaltrials.gov NCT01858376) and materials were approved by The Ohio State University (OSU) Institution Review Board (IRB). All subjects provided written informed consent before participation in the study. Overweight/Class-1 obese (body–mass index [BMI]: 25–35) adult (21–70 years) human subjects of both genders were eligible to participate in this study. Participants were asked to fast overnight before blood sample collections. Any self-reported deviations in diet or exercises were recorded. The subjects were excluded from the study if any one of the following medications was used for management/treatment of CVD-related disorders: beta-blockers, hydrochlorothiazide, statins (Crestor, Lipitor, etc.), aspirin, and ACE inhibitors. The demographics of subjects participating in this study are presented in Table 1. The study design included two baseline visits followed by 12 weeks of supplementation and then 2 weeks of washout. At each visit, 50 mL of blood was collected and the following were recorded: age, sex, height, weight, BMI, blood pressure, and pulse. The blood was used for blinded hospital testing of lipid profile, platelet aggregation, and high-sensitivity CRP.

**Table 1.** Study Subject Demographics

*Parameters*	*Values*	*Baseline*	*12 weeks*
Subjects (*n*)	15		
Age (years)	35.8±3.2		
Gender			
Males	7		
Females	8		
Body weight (lb)		188±7.5	187±7.0
Body–mass index, kg/m^2^		28.8±0.7	28.8±0.6
Blood pressure systolic, mm Hg		117±1.7	117±2.4
Blood pressure diastolic, mm Hg		77.0±1.8	76.0±2.0
Pulse (min)		74.8±3.0	78.4±3.0

Values are expressed as mean±SEM.

### Supplementation regimen and compliance

Each subject received 500 mg of CAPROS (Natreon, Inc., New Brunswick, NJ, USA) supplement b.i.d for 12 weeks. Compliance was monitored by requesting the subjects to return the unfinished or empty bottles to exchange for new ones. Participant supplementation compliance was 83%. Subjects with less than 70% compliance or self-reported noncompliance were excluded from the study.

### Safety monitoring

No serious adverse events were encountered.

### Blood sampling and laboratory methods

At all baseline and follow-up visits, peripheral venous blood was collected in 3.2% sodium citrate tubes to assess lipid profile, hs-CRP, and platelet aggregation. Total cholesterol, high-density lipoprotein cholesterol (HDL-C), low-density lipoprotein cholesterol (LDL-C), triglyceride levels, and CRP were measured using standard clinical lipid profile and CRP test by the Ohio State University Wexner Medical Center Clinical Laboratories. For the measurement of hs-CRP, blood was collected in K_2_ EDTA tubes. Whole blood was centrifuged at 1000 *g* for 15 min at 4°C to separate plasma. Plasma was aliquoted and stored at −80°C. hs-CRP was measured using ELISA (GenWay Biotech, Inc., San Diego, CA, USA) as per the manufacturer's instructions.

### Platelet aggregometry

Platelet function was measured in platelet-rich plasma (PRP) according to the Born^14^ method using an optical aggregometer (Model 700; Chrono-Log Corporation, Havertown, PA, USA). Whole blood was collected by peripheral venipuncture in 3.2% sodium citrate vacuettes (Greiner Bio-One, Monroe, NC, USA). Blood was gently inverted five times to ensure homogeneity. Vacuettes were then allowed to sit for at least 10 min undisturbed at room temperature before preparation of PRP and platelet-poor plasma (PPP) using a PlasmaPrep centrifuge (Separation Technology, Inc., Sanford, FL, USA). Plasma preparations were gently inverted five times to ensure homogeneity. PRP and PPP (500 *μ*L each) were transferred to single-use glass cuvettes for testing. Samples were maintained at 37°C and stirred using silicon-coated stir bars at a speed of 1000 rpm during testing. Following 1 min of stable baseline recording, arachidonic acid (AA; 0.5 mM), adenosine diphosphate (ADP; 10 *μ*M), or collagen (2 *μ*g/mL) agonist were added to PRP. Aggregometry traces were recorded for 9 min following the addition of agonist. After each test, maximum percent aggregation was calculated using AggroLink8 software (Chrono-Log Corporation, Havertown, PA, USA).

### Statistical analyses

Random-effects linear regression models were used to find the association between the clinical outcomes and time due to the longitudinal nature of the data. The model included a fixed term for time and random terms for the slope and intercept. The data were first checked for normality and homoscedasticity over time. Linear contrast statements were used to find mean differences in clinical outcomes at various time points. Each individual clinical outcome was run on a separate regression model. All analyses were run by the study statistician, Gary Philips, using Stata 13.1, (StataCorp, College Station, TX, USA).

## Results

**FIG. 1.**
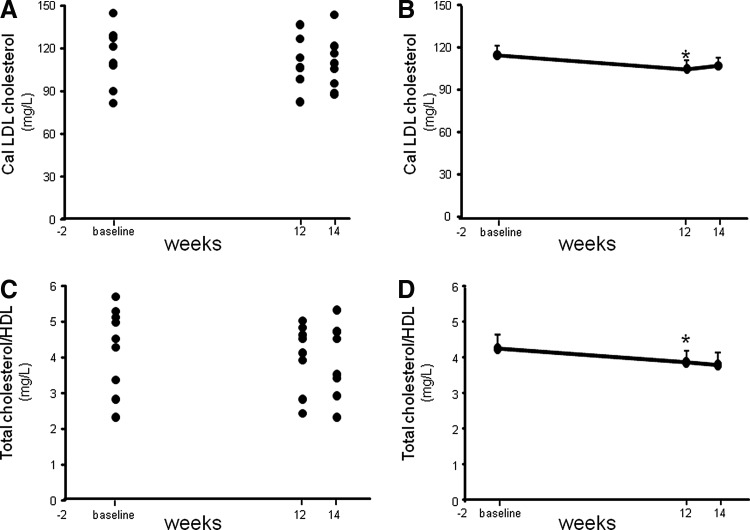
CAPROS^®^ supplementation decreased calculated blood LDL cholesterol and total cholesterol/HDL in overweight/Class-1 obese human subjects. Healthy human subjects were orally supplemented for 12 weeks with CAPROS (500 mg/b.i.d) followed by 2 weeks of washout. Lipid profile was measured in fasting plasma. Calculated LDL cholesterol concentrations plotted individual **(A)** and mean **(B)**. Total cholesterol/HDL plotted individual **(C)** and mean **(D)**. Values are mean±SEM (*n*=9). *denotes *P*<.05 compared to baseline. HDL, high-density lipoprotein; LDL, low-density lipoprotein.

**FIG. 2.**
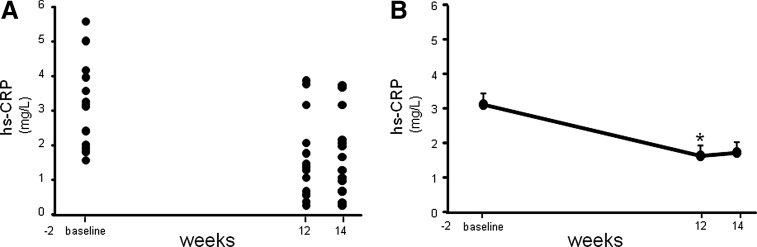
CAPROS supplementation decreases high-sensitivity C reactive protein (hs-CRP) levels in overweight/Class-1 obese human subjects. hs-CRP concentrations were measured in fasting plasma at baseline, 12 weeks (supplementation), and 14 weeks (2 weeks of washout). hs-CRP levels plotted individual **(A)** and mean **(B)**. Values are mean±SEM. *denotes *P*<.05 (*n*=15) compared to baseline.

Fasting lipid profile measurements demonstrated a significant decrease (*P*=.023) in the calculated LDL-C concentration following 12 weeks of supplementation compared to the concentrations observed during averaged baseline visits (Fig. 1 and Table 2). Additionally, a highly significant (*P*=.006) reduction in total cholesterol/HDL was also noted following 12 weeks of CAPROS supplementation (Fig. 1). The concentration of CRP increases when there is systemic inflammation.^15^ A high concentration of CRP is considered a risk factor for heart disease.^15^ A more sensitive CRP test, called a hs-CRP assay, is a promising marker of coronary heart disease.^16^ Although a trend of decreased plasma CRP levels was observed following 12 weeks of supplementation, the effect did not reach significance (*P*=.107). However, a highly significant (*P*<.001) reduction in the hs-CRP levels was noted 12 weeks post-supplementation compared to baseline (Fig. 2).

**Table 2.** Mean Difference Between 12 Weeks Versus Average Baseline and Between 14 and 12 Weeks Using a Random-Effects Linear Regression Model

	*12 vs. 1 baseline*				*14 vs. 12 weeks*			
*Variable*	*Mean*	*95% CI*		P *value*	*Mean*	*95% CI*		P *value*
AA	−14.49	−40.65	11.66	.277	−9.56	−32.15	13.03	.407
ADP	−18.66	−35.62	−1.71	.031	3.34	−12.56	19.23	.681
Collagen	−52.06	−73.28	−30.84	<.001	15.76	−6.86	38.37	.172
Cholesterol	−17.11	−38.10	3.88	.110	8.45	−6.54	23.43	.269
Triglycerides	−0.37	−60.39	59.64	.990	2.82	−38.79	44.43	.894
	−0.96	−15.57	13.66	.898	4.20	−6.21	14.62	.429
HDL cholesterol	−15.91	−29.67	−2.15	.023	3.37	−7.62	14.36	.548
Calculated LDL cholesterol	−0.43	0.74	0.112	.006	−0.06	−0.34	0.22	.662
Total cholesterol/HDL	−16.12	−33.68	1.44	.072	4.23	−8.64	17.10	.519
Non-HDL cholesterol	−16.12	−33.68	1.44	.072	4.23	−8.64	17.10	.519
C-reactive protein	0.51	−1.14	0.11	.107	0.07	−0.44	0.58	.799

CAPROS^®^ supplementation decreased platelet aggregation induced by ADP and collagen. Platelet function was measured at baseline, 12 weeks (supplementation), and 14 weeks (2 weeks of washout).

AA, arachidonic acid; ADP, adenosine diphosphate; HDL, high-density lipoprotein; LDL, low-density lipoprotein.

**FIG. 3.**
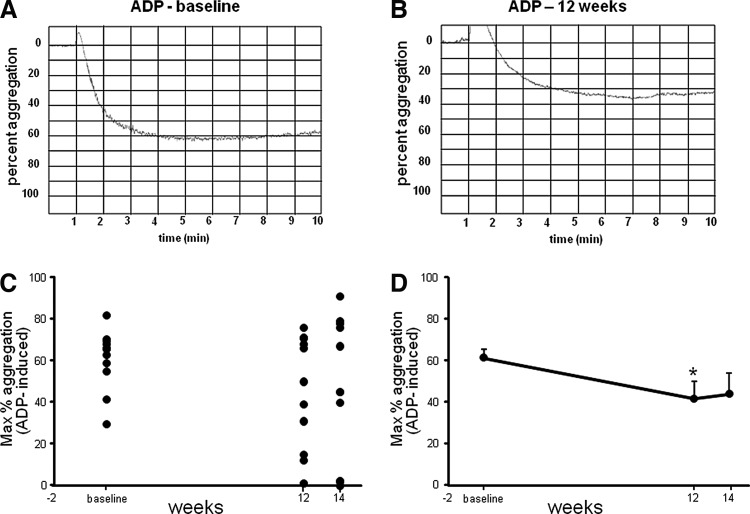
Significant reduction in platelet aggregation induced by adenosine diphosphate (ADP) after CAPROS supplementation. Platelet function was measured at baseline, 12 weeks (supplementation), and 14 weeks (2 weeks of washout). Representative platelet aggregation graphs at baseline **(A)** and after 12 weeks of supplementation **(B)**. The extent of platelet aggregation in response to ADP plotted individual **(C)** and mean **(D)** in platelet-rich plasma (PRP). Values are mean±SEM. *denotes *P*<.05 (*n*=12) compared to baseline.

**FIG. 4.**
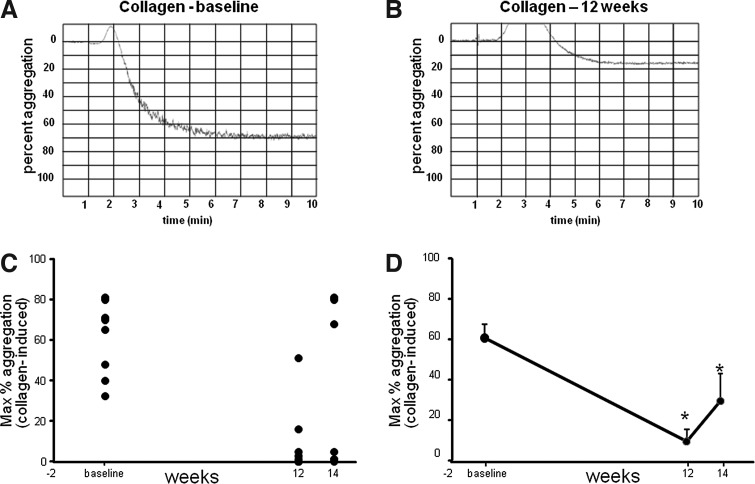
Significant reduction in platelet aggregation induced by collagen after CAPROS supplementation. Platelet function was measured at baseline, 12 weeks (supplementation), and 14 weeks (2 weeks of washout). Representative platelet aggregation graphs at baseline **(A)** and after 12 weeks of supplementation **(B)**. The extent of platelet aggregation in response to collagen plotted individual **(C)** and mean **(D)** in PRP. Values are mean±SEM. *denotes *P*<.05 (*n*=12) compared to baseline.

Platelet hyper-aggregability is associated with the risk factors for CVD. Platelet aggregation was measured in response to ADP, AA, and collagen agonists using an optical aggregometer. The aggregometer software analyzes the traces, reporting the results as maximum amplitude, slope, lag time, and area under the curve. Representative traces from baseline and post-supplementation aggregation measurements have been provided (Figs. 3 and 4). Both ADP- and collagen-induced platelet aggregation was significantly downregulated following 12 weeks of supplementation. For collagen-induced platelet aggregation, the response remained significantly lowered, compared to baseline, even during the washout period, demonstrating a sustained effect.

## Discussion

CVD is a leading cause of death and disability among adults worldwide.^17^ A prospective examination of Framingham Heart Study participants revealed overweight and obesity as major risk factors for the development of CVD.^18^ Hyperlipidemia is recognized as one of the risk factors for CVD.^1,19^ It is established that lowering of serum cholesterol levels leads to a striking reduction in adverse cardiovascular events.^19^ The circulating levels of CRP reflect chronic inflammatory status in humans.^20^ Specifically, hs-CRP is a dependable marker of coronary heart disease.^16^ Platelet aggregation plays an important role in CVD both in the pathogenesis of atherosclerosis and in acute thrombotic events.^21^ Accordingly, increased platelet aggregation is yet another major risk factor for CVD.^22^ The current longitudinal study demonstrates that 3 months of oral supplementation of standardized extract from the fruit of *Phyllanthus emblica*, CAPROS, reduces multiple cardiovascular risk factors in overweight/Class-1 obese adults. A significant decrease in LDL-C, total cholesterol/HDL ratio, hs-CRP, and collagen-induced platelet aggregation suggests that CAPROS supplementation may provide beneficial effects to overweight/Class-1 obese individuals at high risk for CVD.

The cholesterol-lowering effect of CAPROS was comparable to a study where a 1-month supplementation of 50 g of raw amla was found to lower cholesterol and LDL levels in 35 men.^23^ Multiple studies have highlighted the potent antioxidant activity of amla fruit.^7–10^ CAPROS is a standardized aqueous extract of the edible fruits of *Phyllanthus emblica* (amla), containing about 60% of low-molecular-weight hydrolyzable tannins. Using high-performance liquid chromatography (HPLC), the major bioactive tannoids detected were Emblicanin-A, Emblicanin-B, Punigluconin, and Pedunculagin.^4^ Tannoids are naturally occurring plant polyphenols. The cholesterol-lowering activity of CAPROS may be attributed to the polyphenol content of *P. emblica.*^5^ In rodent studies, plant extracts high in polyphenols, such as green tea, have been shown to decrease the solubility of cholesterol in micelles, thereby reducing intestinal cholesterol absorption apparently in response to decreased intracellular cholesterol concentration.^24^ Another recent study demonstrated a significant decrease of LDL-C and a concomitant increase of HDL-C in 42 subjects with type 2 diabetes supplemented with CAPROS (500 mg, twice daily) for 12 weeks.^12,13^

hs-CRP is a robust clinical marker of coronary heart disease.^16^ In a simple regression analysis of 566 healthy subjects, plasma hs-CRP positively correlated with male gender, smoking, BMI, systolic blood pressure, white blood cell count, blood hemoglobin, fasting blood glucose, serum *γ*-GTP, uric acid, and triglycerides. In the same study, plasma CRP was inversely correlated with serum albumin and HDL-C. Variations in circulating hs-CRP levels, even within normal range, are interrelated to the potential of CVD similar to risk factors such as age, smoking, obesity, high blood pressure, and dyslipidemia, which are reported to promote atherosclerosis and ultimately provoke CVD.^16^ The significant hs-CRP-lowering effect observed in the current study is comparable to earlier studies on CAPROS supplementation.^12,13^

In the current study, platelet aggregation was measured following activation with three physiological agonists of platelet activation, specifically AA, ADP, and collagen. AA causes aggregation of platelets through a cyclooxygenase-1 (COX-1)-dependent pathway.^25^ Aspirin strongly inhibits AA-induced platelet aggregation.^25^ Platelets are known to contain high concentrations of ATP, which breaks down during aggregation.^14^ Hellem and Odegaard reported highly specific induction of platelet aggregation by ADP, which is the first breakdown product of ATP.^24^ Collagen is one of the most thrombogenic components of the subendothelium.^26^ Following vascular damage, circulating platelets are exposed to collagen which acts both as a substrate for the adhesion of platelets and as an agonist to platelet activation.^26^ Strong inhibition of collagen-induced platelet aggregation by oral supplementation of CAPROS is a novel finding of this study. Plant extracts rich in polyphenols and tannins have been shown to potently inhibit platelet aggregation.^27^ It is plausible that the inhibition noted in platelet aggregation is because of high polyphenol content of this extract. This finding warrants further investigation into the mechanism of action of CAPROS on ADP- and collagen-induced inhibition of platelet aggregation.

Taken together, this work demonstrates a potent inhibition of collagen-induced platelet aggregation in response to oral supplementation of a standardized extract of *Phyllanthus emblica*. Additionally, the extract significantly inhibited hypercholesterolemia and hs-CRP in overweight/Class-1 obese adult subjects from the US population. Overall, the study demonstrates that oral CAPROS supplementation provides beneficial effects in overweight/Class-1 obese adults by reducing several critical CVD risk factors. Findings of this work warrant a larger placebo-controlled double-blind study to determine the efficacy of this extract on CVD risk factors in overweight/Class-1 obese individuals.

## References

[1] Walden R, Tomlinson B: Cardiovascular disease. In: Herbal Medicine: Biomolecular and Clinical Aspects, 2nd edition, (Benzie IFF, Wachtel-Galor S, eds.). CRC Press, Boca Raton, FL, 2011 22593937

[2] Dasaroju S, Gottumukkala KM: Current trends in the research of Emblica officinalis (Amla): A pharmacological perspective. Int J Pharm Sci Rev Res 2014;24:150–159

[3] Kumar KPS, Bhowmik D, Dutta A, *et al.*: Recent trends in potential traditional indian herbs emblica officinalis and its medicinal importance. J Pharmacognosy Phytochem 2012;1:24–32

[4] Ghosal S: Natural antioxidant compositions, method for obtaining same and cosmetic, pharmaceutical and nutritional formulations thereof. U.S. Patent edUS6124268 A, 2000

[5] Krishnaveni M, Mirunalini S: Therapeutic potential of *Phyllanthus emblica* (amla): The ayurvedic wonder. J Basic Clin Physiol Pharmacol 2010;21:93–105 20506691 10.1515/jbcpp.2010.21.1.93

[6] Singh E, Sharma S, Pareek A, Dwivedi J, Yadav S, Sharma S Phytochemistry, traditional uses and cancer chemopreventive activity of Amla (*Phyllanthus emblica*): The Sustainer. J Appl Pharm Sci 2011;2:176–183

[7] Chatterjee A, Chattopadhyay S, Bandyopadhyay SK: Biphasic effect of *Phyllanthus emblica* L. Extract on NSAID-induced ulcer: An antioxidative trail weaved with immunomodulatory effect. Evid Based Complement Alternat Med 2011;2011:146808 21076542 10.1155/2011/146808 PMC2976071

[8] Chatterjee UR, Bandyopadhyay SS, Ghosh D, Ghosal PK, Ray B: *In vitro* anti-oxidant activity, fluorescence quenching study and structural features of carbohydrate polymers from *Phyllanthus emblica*. Int J Biol Macromol 2011;49:637–642 21741399 10.1016/j.ijbiomac.2011.06.024

[9] Chen TS, Liou SY, Chang YL: Supplementation of Emblica officinalis (Amla) extract reduces oxidative stress in uremic patients. Am J Chin Med 2009;37:19–25 19222108 10.1142/S0192415X09006680

[10] Golechha M, Bhatia J, Arya DS: Studies on effects of Emblica officinalis (Amla) on oxidative stress and cholinergic function in scopolamine induced amnesia in mice. J Environ Biol 2012;33:95–100 23033650

[11] Fatima N, Pingali U, Pilli R: Evaluation of *Phyllanthus emblica* extract on cold pressor induced cardiovascular changes in healthy human subjects. Pharmacognosy Res 2014;6:29–35 24497739 10.4103/0974-8490.122914 PMC3897005

[12] Usharani P, Fatima N, Muralidhar N: Effects of *Phyllanthus emblica* extract on endothelial dysfunction and biomarkers of oxidative stress in patients with type 2 diabetes mellitus: A randomized, double-blind, controlled study. Diabetes Metab Syndr Obes 2013;6:275–284 23935377 10.2147/DMSO.S46341 PMC3735284

[13] Usharani P, Kishan PV, Fatima N, Kumar UC: A comparative study to evaluate the effect of highly standardised aqueous extracts of *Phyllanthus emblica*, withania somnifera and their combination on endothelial dysfunction and biomarkers in patients with type II diabetes mellitus. Int J Pharm Sci Res 2014;5: 2687–2697

[14] Born GV, Cross MJ: The aggregation of blood platelets. J Physiol 1963;168:178–195 14056485 10.1113/jphysiol.1963.sp007185 PMC1359417

[15] Vasan RS: Biomarkers of cardiovascular disease: molecular basis and practical considerations. Circulation 2006;113:2335–2362 16702488 10.1161/CIRCULATIONAHA.104.482570

[16] Saito M, Ishimitsu T, Minami J, Ono H, Ohrui M, Matsuoka H: Relations of plasma high-sensitivity C-reactive protein to traditional cardiovascular risk factors. Atherosclerosis 2003;167:73–79 12618270 10.1016/s0021-9150(02)00380-5

[17] WHO (World Health Organization): Preventing Chronic Disease: A Vital Investment. WHO Press, 2007 www.who.int/chp/chronic_disease_report/contents/en/

[18] Wilson PW, D'Agostino RB, Sullivan L, Parise H, Kannel WB: Overweight and obesity as determinants of cardiovascular risk: The Framingham experience. Arch Intern Med 2002;162:1867–1872 12196085 10.1001/archinte.162.16.1867

[19] Lacoste L, Lam JY, Hung J, Letchacovski G, Solymoss CB, Waters D: Hyperlipidemia and coronary disease. Correction of the increased thrombogenic potential with cholesterol reduction. Circulation 1995;92:3172–3177 7586300 10.1161/01.cir.92.11.3172

[20] Visser M, Bouter LM, McQuillan GM, Wener MH, Harris TB: Elevated C-reactive protein levels in overweight and obese adults. JAMA 1999;282:2131–2135 10591334 10.1001/jama.282.22.2131

[21] Furie B, Furie BC: Mechanisms of thrombus formation. N Engl J Med 2008;359:938–949 18753650 10.1056/NEJMra0801082

[22] Willoughby S, Holmes A, Loscalzo J: Platelets and cardiovascular disease. Eur J Cardiovasc Nurs 2002;1:273–288 14622657 10.1016/s1474-5151(02)00038-5

[23] Jacob A, Pandey M, Kapoor S, Saroja R: Effect of the Indian gooseberry (amla) on serum cholesterol levels in men aged 35–55 years. Eur J Clin Nutr 1988;42):939–944 3250870

[24] Hellem AJ, Odegaard AE: Investigations on adenosine diphosphate (Adp) induced platelet adhesiveness in vitro. I. The Adp-platelet reaction in various experimental conditions. Thromb Diath Haemorrh 1963;10:61–70 14081291

[25] Silver MJ, Smith JB, Ingerman C, Kocsis JJ: Arachidonic acid-induced human platelet aggregation and prostaglandin formation. Prostaglandins 1973;4:863–875 4205973 10.1016/0090-6980(73)90121-4

[26] Baumgartner HR, Haudenschild C: Adhesion of platelets to subendothelium. Ann N Y Acad Sci 1972;201:22–36 4346061 10.1111/j.1749-6632.1972.tb16285.x

[27] Mattiello T, Trifiro E, Jotti GS, Pulcinelli FM: Effects of pomegranate juice and extract polyphenols on platelet function. J Med Food 2009;12:334–339 19459734 10.1089/jmf.2007.0640

